# Public Social Media Discussions on Agricultural Product Safety Incidents: Chinese African Swine Fever Debate on Weibo

**DOI:** 10.3389/fpsyg.2022.903760

**Published:** 2022-05-20

**Authors:** Qian Jiang, Ya Xue, Yan Hu, Yibin Li

**Affiliations:** ^1^School of Geography and Resource Science, Neijiang Normal University, Neijiang, China; ^2^Neijiang Center for Disease Control and Prevention, Neijiang, China; ^3^School of Economics and Management, Neijiang Normal University, Neijiang, China; ^4^Tuojiang River Basin High-Quality Development Research Center, Neijiang, China

**Keywords:** agricultural product safety incidents, African swine fever, social media, information dissemination, diffusion patterns

## Abstract

Public concern over major agricultural product safety incidents, such as swine flu and avian flu, can intensify financial losses in the livestock and poultry industries. Crawler technology were applied to reviewed the Weibo social media discussions on the African Swine Fever (ASF) incident in China that was reported on 3 August 2018, and used content analysis and network analysis to specifically examine the online public opinion network dissemination characteristics of verified individual users, institutional users and ordinary users. It was found that: (1) attention paid to topics related to “epidemic,” “treatment,” “effect” and “prevent” decrease in turn, with the interest in “prevent” increasing significantly when human infections were possible; (2) verified individual users were most concerned about epidemic prevention and control and play a supervisory role, the greatest concern of institutional users and ordinary users were issues related to agricultural industry and agricultural products price fluctuations respectively; (3) among institutional users, media was the main opinion leader, and among non-institutional users, elites from all walks of life, especially the food safety personnel acted as opinion leaders. Based on these findings, some policy suggestions are given: determine the nature of the risk to human health of the safety incident, stabilizing prices of relevant agricultural products, and giving play to the role of information dissemination of relevant institutions.

## Introduction

The last decade had seen several public health food safety incidents, such as swine flu in North America in 2009 (Garten Rebecca et al., 2009), avian influenza in China in 2013 (Gao et al., 2013) and the African swine fever (ASF) outbreak in 2018 (Zhou et al., 2018), all of which directly impacted the livestock and poultry industries and raised public health concerns over food safety, which in turn indirectly led to price volatility in pork, eggs, and other related agricultural products (Chmielewski and Swayne, 2011; Hassouneh et al., 2012; Wen et al., 2019; Yi et al., 2019b). ASF, a highly contagious viral hemorrhagic disease of swine, appeared and spread in most parts of the world, with significant economic implications for the pig industry and pork prices (Zhao et al., 2022a). As China is the world’s largest pork producer and consumer, accounting for about 50% of the global pork supply, ASF had a huge impact on China’s agricultural economy (Muñoz and Tabarés, 2022). From October 2018 to September 2019, pork prices rose 85.7% year-on-year in China (Xu et al., 2022). Effective prevention and control of the epidemic is an inevitable trend to ensure sustainable social and economic development.

Extensive media and social media coverage quickly made the public aware of these major agricultural product safety incidents and played a significant role in fanning the flames and expanding the economic impacts (Wen et al., 2019; Yi et al., 2019b). In the past, information about agricultural product safety incidents was mainly accessed from traditional media, such as newspapers, radio, and television (De Jonge et al., 2010; Attavanich et al., 2011; Carducci et al., 2011; Hilton and Hunt, 2011; Vasterman and Ruigrok, 2013); however, the rise in social media means that people are no longer unilaterally receiving messages from official organizations but are participating in the information dissemination (Neuman et al., 2014).

Due to its ease of use, timeliness and openness, Twitter has become a major global platform for spreading information and expressing opinions (Simon et al., 2015). However, in China, Weibo, which is similar to Twitter, is the most widely used social media (Fung et al., 2013; Song et al., 2020), with 462 million monthly active users in 2018 and 130 million, 120 million, and 1.5 million average daily texts, pictures, and video/live streams (*The 2018 Weibo User Development Report*). Therefore, the latest news on agricultural product safety incidents can be rapidly spread on Weibo; however, the excessive spread of unverified news and rumors can result in panic and huge economic losses (Gu et al., 2014; Martínez-Rojas et al., 2018; Stieglitz et al., 2018). The ASF epidemic in China has caused huge online discussion, causing a great deal of misinformation and panic. Timely understanding online public discussion topics and communication characteristics in ASF events is not only of great significance for relevant institutions to effectively guide and control public opinions but also provides targeted directions for epidemic prevention and control.

Therefore, because it was a recent Chinese food safety and public health incident, the ASF Weibo content from 2 August 2018 to 2 August 2019 was studied to: (1) analyze the discussion and ASF information sharing by different Weibo user types; and (2) analyze the social network structures of the Weibo users discussing the ASF.

The remainder of this paper is organized as follows: section Literature Review reviews the online public opinion research on agricultural product safety incidents, section Research Background gives the background to the ASF outbreak, section Research Design and Method details the research steps and methods, section Results and Discussion discusses the analysis results, and section Conclusions and Recommendations gives the conclusions and recommendations.

## Literature Review

The agricultural product safety social media discussions were summarized under three main headings: content analysis of social media discussions; network analysis of social media discussions; and the main social media discussion subjects/stakeholders.

### Content Analysis of Social Media Discussions

Social media data are mostly unstructured textual data that need to be converted into structured data for analysis (Lipizzi et al., 2015; Aswani et al., 2018), with most previous studies having adopted manual content analyses that divided the text content into predetermined types using manual coding (Collier et al., 2010; Ding and Zhang, 2010; Gao et al., 2011; Tirkkonen and Luoma-aho, 2011; Freberg et al., 2013; Luoma-Aho et al., 2013). For example, Chew and Eysenbach (2010) analyzed the 2009 swine flu Twitter discussions using manual content analysis and divided the tweets into several types, such as resources, personal experience, personal opinions, and interests. However, this approach can be relatively subjective and the number of tweets that can be analyzed is limited. Computerized automatic content analyses can also be employed to assess the topics, sentiments, and other content in large amounts of text, with the most common methods being topical analysis, semantic network analysis, sentiment analysis, and machine learning methods (Krippendorff, 2004) such as support vector machines (SVM) and Naive Bayes (Lyons and Lăzăroiu, 2020; Mircică, 2020; Stanley and Kucera, 2021). Content analysis of social media is often applied in the analysis of travel decisions. Pop et al. (2022) showed that consumers’ trust in social media influencers had a positive impact on all stages of travel decision-making. Hou et al. (2019) extracted subject terms from reviews of three major online travel agencies in China and constructed semantic association networks to distill five aspects of tour guides, hotels, services, scenic spots, and experiences that were of concern to tourists. All these studies have reflected the importance of social media for travel decisions, and tourism companies, online travel agencies, and hotels could use social media to guide consumption decisions and achieve greater benefits.

In addition, text mining has often been applied to the study of social media discussions of emergencies. Kim et al. (2018) conducted a textual analysis of Twitter content before and after Storm Cindy in the United States in 2017 and found that negative words such as “threatening” and “scary” appeared more frequently after the event than before it, thus assuming that evolving words over time can suggest the extent of the event. Pourebrahim et al. (2019) studied Twitter texts before, during, and after Hurricane Sandy and further conducted a sentiment analysis, reaching similar conclusions. Furthermore, their analysis of word co-occurrence revealed that during disasters, emotional tweets are increasingly used to release anxiety and provide a sense of connection between people experiencing similar situations, while relief and donation-related clusters become major topics of discussion in the aftermath of disasters. In response to public health events, Hellsten et al. (2019) examined Twitter discussions on avian influenza in the Netherlands from 2015 to 2017, applying automated network analysis to examine the co-occurrence of passive stakeholders and thematic networks. In particular, the global pandemic of COVID-19 since 2020 has been followed by social media discussions during the epidemic. Some studies have focused on social media discussions of specific issues during the outbreak, including willingness to vaccinate (Griffith et al., 2021; Hussain et al., 2021) and discussions of older adults and COVID-19 (Jimenez-Sotomayor et al., 2020; Xiang et al., 2021). Others were concerned about topics of public concern and changes in sentiment in social media discussions, and these studies helped government and public health agencies to better communicate with the public, stabilize public sentiment, and combat the spread of fake news (Abd-Alrazaq et al., 2020; Han et al., 2020; Zhao et al., 2020; Jang et al., 2021).

### Network Analysis of Social Media Discussions

Social media communication patterns have also attracted significant recent research attention (Nair et al., 2017; Kim et al., 2018; Vijaykumar et al., 2018), with the nodes in a social media network representing the users and the connections, such as following, commenting, reposting or retweeting, representing the user relationships. Centrality has also been highlighted as an important social network concept, with the higher the centrality, the more important the node’s role in the network, that is, users with high centrality generally attract higher social media network attention. For example, Kim et al. (2018) used social network analysis to investigate the information network and diffusion during Hurricane Cindy in the United States in 2017 and found that news and meteorological agencies were the main information sources, while the public and organizations were the main information disseminators. Similarly, Pourebrahim et al. (2019) studied Twitter discussion of Hurricane Sandy and found that the connections between key influencers from different fields such as politics, news, weather and relief organizations and their followers played a crucial role in information sharing and dissemination, which provide an effective tool for emergency managers to establish two-way communication during disasters. Closely related to this study, Sun et al. (2020) similarly studied the information dissemination patterns of Weibo during the African swine fever in 2018 and the results showed that different types of users play different roles, such as providing information, amplifying information, delivering information and engaging other users, and its beneficial for government agencies and public health authorities to effectively disseminate information.

### Agricultural Product Safety Incident Social Media Subjects/Stakeholders

Social media discussion research on emergencies/crises often involve three main subjects: the government, the network media and the netizens. Emergencies/crises tend to generate public sentiment, with the network media promoting the discussion development; however, at the same time, the government attempts to supervise and actively guide public sentiment (Li et al., 2020). Therefore, research into the different subjects/stakeholders can elucidate various responses. Past research has tended to adopt manual coding to identify social media subjects/stakeholders. For example, Hellsten et al. (2019) divided 122 Twitter users into media, citizens, environmental organizations, conventional industries, eco-industries, public organizations, political actors, and others using manual coding. However, manual coding has a certain subjectivity and can only be used if the number of users is relatively small; therefore, automatic coding has been found to be more scientific and efficient for large amounts of data. As Weibo authentication divides users into institutional users (official), verified individual users (well-known figures) and unverified individual users (ordinary users), the information can be easily divided into three groups when information mining.

To sum up, first of all, the existing studies on social media discussion of emergencies mostly focus on disaster events. Since the outbreak of COVID-19, public health events have attracted increasing attention. However, there are still few studies on agricultural product safety events. Therefore, this study focuses on the social media discussion of African Swine fever (ASF) in China, an agricultural product safety event, based on Weibo platform. Secondly, existing social media discussions on agricultural product safety incidents have tended to adopt manual content analysis, which had resulted in problems such as small sample sizes and subjective classifications. In this study, automatic coding was applied using information mining on the Weibo ASF related users, as well as automatic content analysis methods such as topic analysis, semantic network analysis, and sentiment analysis were applied to study the ASF discussion developments in the three user groups. Finally, in addition to the estimation and prediction of cases through social media data (Signorini et al., 2011; Chen et al., 2019; Masri et al., 2019), there was a lack of research into the social media propagation patterns of such incidents. From the perspective of communication network, a network analysis method was applied to study the ASF public opinion network dissemination characteristics and identify the opinion leaders and dissemination bridges.

## Research Background

**Figure 1:**
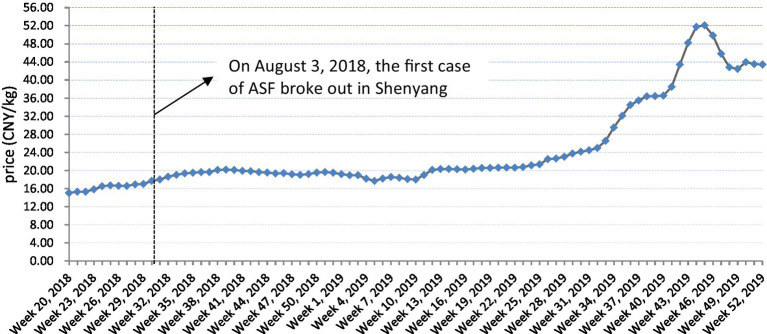
Changes in pork prices in mainland China since May 2018. Data source: China animal husbandry information network.

Since the first case of ASF was detected in Shenyang, Liaoning province on 3 August 2018, it continued to spread in China, with 75 cases identified by the end of 2019 in all of China’s 31 mainland provinces/autonomous regions/municipalities. Because of its high lethality, from the initial outbreak, ASF has had a significant impact on the market price of pigs and related products in China (Hu and Guo, 2018). The price trends for dressed pork nationwide since May 2018 are shown in Figure 1.

As can be seen, during the initial ASF outbreak, there was no significant change in the national pork prices, which was probably because of regional short-term price fluctuation differences. Farmers in the main live pig producing areas sold a great deal of their stock to avoid the risks, with this sudden oversupply leading to price decreases; while the prices in the main sales areas for live pigs increased because of transportation restrictions. However, over the longer term, pork prices in late 2019 were about three times as high as before the ASF outbreak because of the lack of supply and the pull of demand.

**Figure 2:**
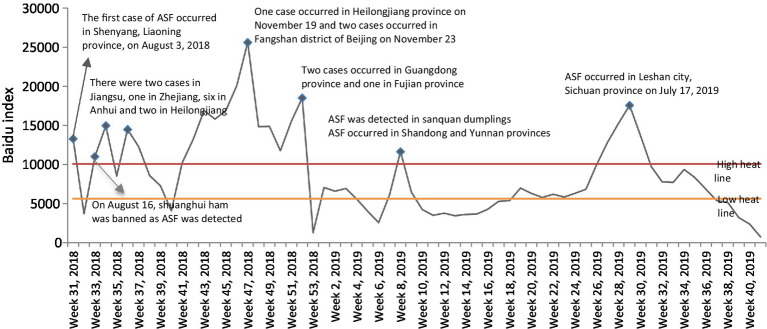
Search-term interest during the ASF outbreak in China.

The Baidu index (like google search index), which reflects the number of Baidu searches, can be used to measure public interest in the ASF outbreak (Gu et al., 2014; Towers et al., 2015; Yi et al., 2019a). The results from a search-term index (keyword: African swine fever) from 2 August 2018 to 2 August 2019 were examined and the events corresponding to each peak identified, as shown in Figure 2.

## Research Design and Method

**Figure 3:**
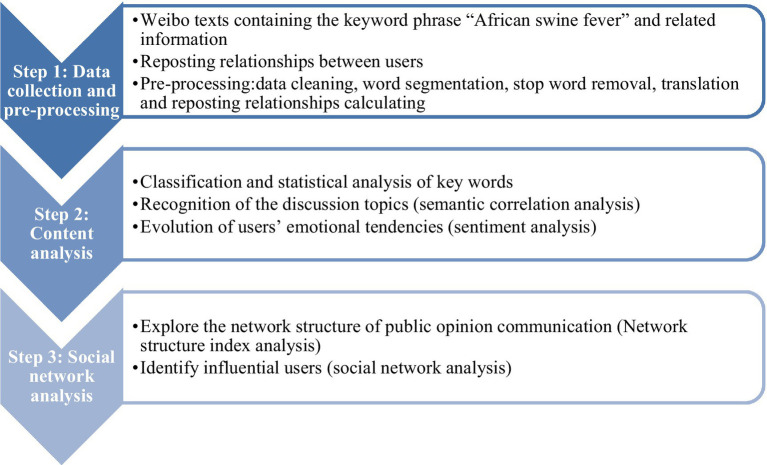
Framework for the ASF social media discussions.

In order to fully grasp the online public discussion and communication characteristics on ASF, to implement more targeted public opinion guidance and control measures and maintain a healthy social network environment, content analysis, and network analysis methods were applied to online public opinion content to identify the ASF public opinion transmission characteristics. Figure 3 summarizes the study research process. First, a web crawler to identify ASF-related content in Weibo and Weibo-reposting-network. Then, pre-processed textual data: data cleansing, tokenization, stop word removal, and translation: using Jieba, the Python Chinese word segmentation natural language processing (NLP) module (Hou et al., 2019), after which classified subject words and statistically analyzed, semantic association analysis, and sentiment analysis performed on the processed textual data, and social network analysis performed on the reposted network data.

### Data Collection and Pre-processing

#### Data Collection

To analyze the online public discussion contents and the public opinion communication network structure, data need to be collected from the following two parts: Weibo texts containing the key “African swine fever” phrase; and user network data from the reposting of the Weibo posts.

##### Search “African Swine Fever” Weibo Post Information

To compare the differences between different user groups in the discussion focus on ASF events, the discussion content, users participating in the discussion and relevant information were collected. First, temporal logic was used to search for the original Weibo African swine fever keyword information and acquire the corresponding microblog content addresses. The time period was set from 2 August 2018, when the first ASF occurred in China, to 2 August 2019. Then, a public free Python Crawler Technology was used to automatically collect the information from the web pages and extract the microblog information: poster ID, authentication information, repost number, content, release date, poster’s gender, location, and other information.

##### Collect the Weibo Reposting Relationships

To explore the status and role of different user groups in online public opinion communication, reposting relationships data were collected. If all reposted posts were collected, there was a high possibility that most would have come from a discussion high heat period. Therefore, to ensure the data reliability, first, the search indexes were ranked from large to small, with the first third of the search indexes corresponding to discussion high heat periods and the last third corresponding to discussion low heat periods (Figure 2). Based on the proportion of the total Weibo posts in each period, 25, 8, and 4 microblogs were, respectively, randomly selected from the top 3% Weibo reposted posts in the discussion high, medium, and low heat periods. Then, the Python crawler technology was allowed to retrieve the original posters’ IDs and their re-posts (Bird et al., 2009).

#### Data Pre-processing

It is necessary to pre-process data to obtain standard-format data for further analysis because the collected data is often cluttered and nonstandard (Martínez-Rojas et al., 2018; Song et al., 2020). On the one hand, the Weibo posts were divided into three categories based on the origins, after which each category was pre-processed over four steps: data cleaning, word segmentation, stop word removal and translation. First, the purpose of data cleansing is to filter out useless information to reduce its impact on analysis results. After the data cleaning, 82,203 Weibo posts were collected: 12,659 from verified individual users; 21,015 from institutional users; and 48,529 from ordinary users. Second, as Chinese has no obvious blanks in sentences (Shuang et al., 2018), word segmentation was then required on the collected Weibo texts. Jieba, the most widely used Chinese word segmentation component, was used to implement the tokenization and stop word removal, which are words that plays a cohesive role in a sentence and has no real value to the text meaning. Finally, the first 200 Chinese keywords for each kind of user were selected and translated into English. On the other hand, the reposting relationships between users were calculated. For example, once A reposted B’s post, a line with a weight of 1 was created pointing from B to A. Therefore, the many reposting relationships between the Weibo users constituted the public opinion communication network. To avoid multiple calculations, only direct reposts were calculated. For example, in A → B → C relation, A → B, B → C direct reposts were calculated, while A → C indirect repost was not counted.

### Content Analysis

The purpose of content analysis is to identify the focus and emotional changes of online users on ASF events. Content analysis method, which is an extensive set of natural language processing (NLP) and text mining techniques (Chau and Xu, 2012), was used to analyze “unstructured” text data from the perspective of topic analysis and sentiment analysis and to compare the differences in discussion and sentiment among different user groups.

#### Topic Analysis

The purpose of topic analysis is to reveal the different user groups discussion focus. The first part of the ASF Weibo data analysis was to identify the thematic words, which were the concise, timely and informative words that had certain meanings (Hou et al., 2019), and apply the thematic analysis to calculate the keyword frequencies in the texts. The second part of the ASF Weibo data analysis was the semantic association analysis. This study used Bigrams in NLTK Chinese corpus to generate semantically related binary co-occurrence phrases. After tokenization, the binary co-occurrence frequencies of the adjacent words from the three users’ microblog texts were, respectively, calculated to identify the key phrases and the main discussion topics (Chae, 2015).

#### Sentiment Analysis

The purpose of sentiment analysis is to reveal the sentiment evolution trend of different user groups. The subjective information in Weibo posts was classified (Feldman, 2013) and each post was classified as negative, neutral, or positive. While there are different techniques and tools for sentiment analysis (Pang and Lee, 2008), SentiStrength was chosen as the sentiment analysis algorithm because it was originally designed for informal texts such as tweets (Thelwall et al., 2011). Therefore, every post sentiment was measured, with scores greater than 0 representing positive emotions, those equal to 0 representing neutral emotions, and those less than 0 representing negative emotions.

### Social Network Analysis

In order to guide network users’ communication behavior, social network analysis is necessary. Social network analysis, which is based on mathematical methods and graph theory (Otte and Rousseau, 2002; Prell, 2012) and can identify the position and role of different user groups in public opinion communication according to the reposting relationship between users, was specifically developed to construct ASF public opinion communication network, reveal network structure, and visualize these network relationships using software programs such as “Ucinet” and “Gephi,” thereby allowing for an intuitive analysis of the node behavior and a deeper understanding of the network structure. Various network metrics provide a detailed description of the network, the specific descriptions and calculation methods for which are shown in Table 1.

**Table 1:** Specific SNA indicators.

Assessing Content	Parameter	Determination	Formula	Note
Whole network analysis (Freeman et al., 1979; Assenov et al., 2007)	Diameter	The maximum distance between any two nodes in the network.		
	Ave. path length	The number of edges on the shortest path connecting any two nodes.	$APL=\frac{\sum d_{ij}}{n\left(n-1\right)}$	$d_{ij}$: the shortest path between node i and j
Individual network analysis (Gan et al., 2018; Yuan et al., 2018)	Degree centrality	Degree centrality is measured by the number of nodes reposting the user or reposted by the user, and can be divided into In-degree and Out-degrees depending on the relationship directions. In-degree is the initiative of the user to interact with other nodes and the Out-degree indicates the extent to which a user is recognized by other nodes.	$DC\left(n_{i}\right)=\deg\left(n_{i}\right)$	$\deg\left(n_{i}\right)$: the number of nodes connected with node $n_{i}$
	Betweenness centrality	Betweenness centrality measures the ability of a user to control the communication of other nodes.	$BC\left(n_{i}\right)=\frac{2\overset{N}{\underset{j}{\sum }}\overset{N}{\underset{k}{\sum }}b_{jk}\left(i\right)}{N^{2}-3N+2}$	$b_{jk}\left(i\right)$: the probability of a third user i falling on the shortcut between user j and k.

## Results and Discussion

### General Overview

**Figure 4:**
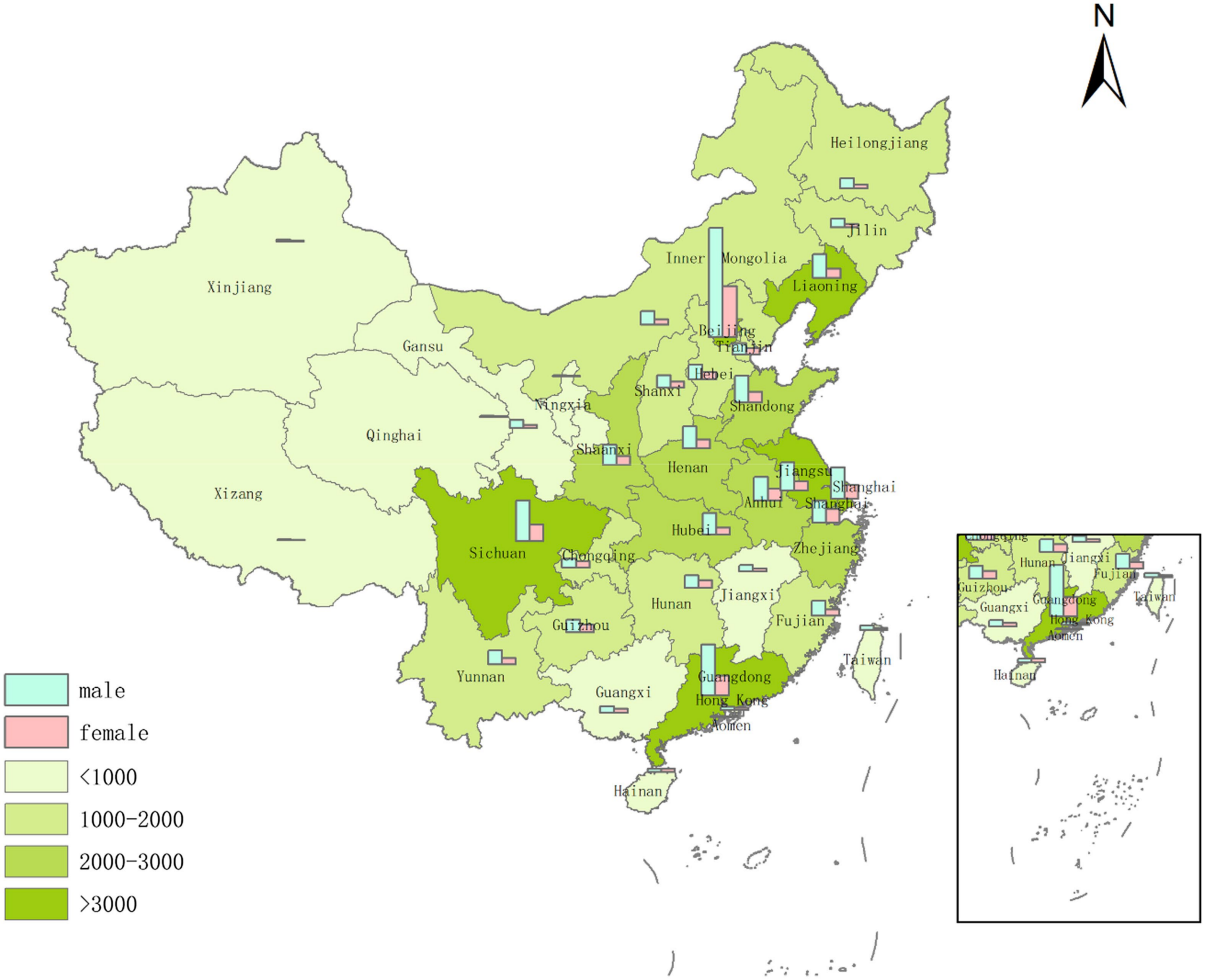
Gender and regional distribution of users participating in the ASF-related discussions.

The number of posts reflect the public attention on the incident (Mollema et al., 2015; Househ, 2016). However, as gender differences could have a significant impact on attention to public health events, and the number of posts from different regions reflects the regional discussion heat, the collected gender and address information of the 82,203 Weibo posters was used to determine the gender differences and the ASF discussion concentrations. As shown in Figure 4, the number of Weibo discussion users decreased from east to west. As the eastern region was more involved in the discussion region, Beijing, Liaoning, Sichuan, Guangdong, Jiangsu, and Shanghai were placed on the first level of discussion heat. Because of the more developed economies, the denser populations and the more advanced information and communication technologies in the eastern coastal region, data transmission is generally faster, which allows more information to be disseminated (Sepehrdoust, 2018). Beijing had the greater ASF discussion volume, possibly because it is the capital of China and has many official registered Weibo accounts, which meant that great deal of ASF information was generated from here. However, there were also dense discussions in Liaoning, where the first ASF case in China was confirmed on 3 August 2018, and in Sichuan, which is a major pig breeding province and also one of the core pork supply areas. On 15 November 2018, the first ASF case in Sichuan was recorded, which had a significant impact on the pig industry and severely affected pork prices.

It was also observed that the number of Weibo posts by male users was significantly higher than posts by female users (Strekalova, 2015). There were several possible reasons for this finding. First, the 2018 Weibo user development report released by the Sina Weibo data center reported that there was a higher proportion of male Weibo users than female users. Second, men tend to have more confident, dominant discourse styles, while women are more cooperative and supportive (Hayat et al., 2017), which means that men are more likely to express their own opinions through their original posts, while women would tend to express their feelings more frequently in comments. These results, however, were contrary to Xie et al. (2017), possibly because males and females have different online discussion participation levels for different types of emergencies, that is, women are more likely than men to express their opinions on social media due to their strong sympathy for catastrophic events that cause harm to people’s lives (Liu et al., 2020).

### Involvement of Different User Groups in the Discussion: Content Analysis

The content analysis addressed the following four research questions: (1) What were the keywords discussed and what were the keyword differences between the three user types? (2) What were the main topics discussed by different user types? (3) What were the similarities and differences between the topics discussed during the ASF and similar public health events? and (4) What were the emotional tendencies of different users and how did they change over time?

#### ASF Online Public Opinion Discussion Topic Analysis

##### Keyword Classification and Statistical Analysis

Word frequency is used to measure the importance of words in the text, with higher frequency words being more likely to represent issues of concern (Lam et al., 2019). As simple statistical key word frequency analyses are unable to explain the meaning of the words, the keywords in the previous studies (Corrales-Garay et al., 2019) have often classified epidemic related online public opinion into categories such as health risks, prevention, symptoms, transmission and treatment (Vijaykumar et al., 2018; Khatua et al., 2019). Therefore, in this study, the context factors were taken into account and four different categories identified: prevention, epidemic, disposal, and effect. First, the keywords from the three user groups were combined and the top 500 highest frequencies selected. However, “African swine fever” and words of the same or similar meaning were not included as these referred to the event itself and did not require any specific classification. Second, the key word classification was conducted simultaneously and independently by two graduate student groups familiar with the ASF event, after which the classification results were compared, discussed and finally determined, as shown in Table 2, with the associated value representing the word frequency as a percentage of the total frequency.

**Table 2:** Classification and statistics for the key words.

Prevention		Epidemic		Disposal		Effect	
Prevention and control	0.91%	Epidemic	2.50%	Ministry of Rural Affairs	1.20%	Pork	1.11%
Work	0.39%	Pig	1.41%	Block	0.53%	Agriculture	0.58%
Disinfect	0.14%	Occur	0.82%	Cull	0.50%	Food	0.28%
Safety	0.14%	Virus	0.74%	Harmless	0.44%	Livestock on hand	0.26%
Examine	0.12%	Epidemic disease	0.45%	Check	0.35%	Farm	0.19%
Control	0.12%	Shenyang	0.36%	Release	0.33%	Food safety	0.17%
Control center	0.11%	Death	0.36%	LBVD	0.23%	Stock	0.16%
Anti-epidemic	0.11%	Epidemic area	0.34%	Dispose	0.21%	Concept stock	0.15%
Ban	0.11%	Infect	0.33%	Slaughter	0.18%	Price	0.12%
Science popularization	0.11%	Sanquan	0.32%	Slaughter house	0.14%	Effect	0.12%

As can be seen, the public were most concerned about the “epidemic” topic in the ASF event, among which “epidemic” and “pig” accounted for more than 1%, and “occur” and “virus” accounted for more than 0.5%, which indicated that these keywords were the most discussed. For the two disposal and effect topics, the “Ministry of Rural Affairs” and “pork” were the most popular words with public ratios of 1.20% and 1.11%, which indicated that the response and handling measures by the Ministry of Rural Affairs toward pork safety and pricing were of public concern. To study the differences between the ASF social media discussions and other agricultural product safety incidents, the Weibo data related to this avian influenza in 2017 was mined and analyzed in the same way. The difference is that the avian influenza was most focused on “infect” and “case.” In contrast to the ASF, the avian influenza users paid less attention to the “effect” theme than to the “prevent” theme, possibly because ASF is not a zoonotic disease, which meant that users were more concerned about the living pigs being infected as well as the price of pork and other livelihood issues. However, avian influenza is a zoonotic disease that has a high death rate in humans (Mcaleer et al., 2010) and therefore requires strict prevention measures such as vaccines.

To better understand and compare the distribution differences between the four topics in the three user groups, the high-frequency words from the three user types were statistically analyzed. The threshold values for the high-frequency words were calculated based on Donohue (1973) classification method for high-frequency and low-frequency words, the formula for which is $T=-1+\sqrt{1+8I_{1}}/2$, where *T* is a boundary value for the high-frequency and low-frequency words, which is also known as the threshold value for the high-frequency words and represents the frequency of the last word in the high-frequency words, and $I_{1}$ is the number of keywords with a frequency of 1. The calculation results are shown in Table 3, from which it can be seen that the threshold values for the high-frequency words for the verified individual users, institutional users and ordinary users were 155.03, 153.23, and 240.76, respectively, and the corresponding high frequency words were 151, 226, and 351.

**Table 3:** Threshold statistics for the high-frequency words.

Users	Number of keywords	$I_{1}$	T	Number of high-frequency words
Verified individual users	22,424	12,173	155.03	151
Institutional users	22,595	11,894	153.23	226
Ordinary users	56,913	29,223	240.76	351

**Figure 5:**
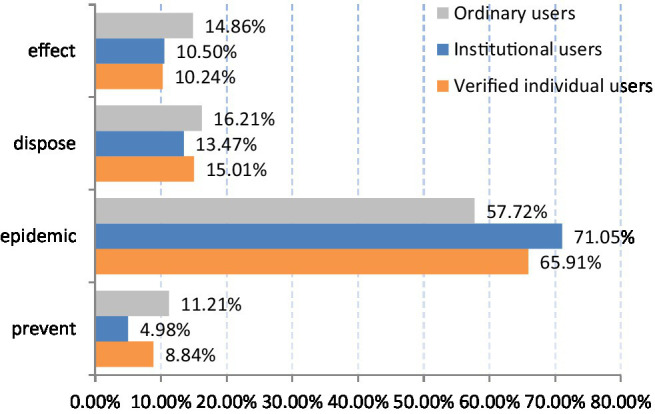
Distribution of the four topics in the three user groups.

Based on the calculated threshold values for the high-frequency words, the distribution differences for the four topics by the three user groups were determined, as shown in Figure 5, in which the horizontal axis represents the percentage of high-frequency words for the four topics in the three user groups; the larger the percentage, the more attention the topic received; and the vertical axis represents the four topics that users paid most attention to.

As can be seen, the three-user group discussion topics were similar. First, the “epidemic” percentage was significantly higher than the other three topics at greater than 50%, which indicated that all three user groups were most concerned about the epidemic itself and the control of the development and spread of the epidemic by the relevant institutions (Vijaykumar et al., 2018). Second, the “effect,” “disposal” and “prevention” percentages were relatively low, with “effect” and “disposal” being only slightly higher than “prevent.” This may have been because the sudden first occurrence of the epidemic meant that no prevention work had been carried out on time, and also because as the ASF virus impacted pigs rather than people, less attention was paid to prevention work. The results also indicated how quickly and effectively the correct information was being disseminated compared to the Twitter content swine flu discussions in 2009, which revealed that Twitter users at that time believed that pigs and pork could host and/or transmit the virus (Ahmed et al., 2019).

The topic distribution varied slightly between the three user groups. The distributions for “effect” and “disposal” in the three user groups were similar, but there were some differences in the “epidemic” and “prevention” distributions. The “epidemic” percentage by institutional users (71.05%) was significantly higher than by ordinary users (57.72%), which indicated that the institutional users paid more attention to the ASF virus pig infection morbidity and death rate than the ordinary users, possibly because the institutional users were worried about the effects on China’s pig industry. Compared with the ordinary users (11.21%) and the verified individual users (8.84%), the “prevention” topic was of little concern to the institutional users (4.98%), which was in sharp contrast to their focus on the “epidemic.”

##### Semantic Correlation Analysis

1. Construct semantic association co-occurrence phrases
  Words appearing simultaneously in Weibo texts possibly have some correlations. It is difficult to accurately grasp the public discussion real focus and the internal connection and correlation degree of each discussion topic simply by statistics of text subject words. If two words often appear together in different texts, it indicates that these texts have some common characteristics, with the higher the co-occurrence frequency, the higher the correlation degree. To explore the relationships between the keywords, the semantic association co-occurrence phrases were constructed for the three user types from which 210, 220 and 750 thousand co-occurrence phrases were, respectively, identified for the verified individual users, the institutional users, and the ordinary users. To better analyze the semantic correlations between the keywords, the top 200 co-occurrence phrases, all of which had over 40 co-occurrence frequencies, for each topic were selected. The top 10 co-occurrence phrases for each topic are shown in Table 4.
  **Table 4:** Top 10 semantic association co-occurrence phrases.

Verified individual users		Institutional users		Ordinary users	
Bigram phrases	Weight	Bigram phrases	Weight	Bigram phrase	Weight
Africa–swine fever	27,549	Africa–swine fever	45,786	Africa–swine fever	98,258
Swine fever–epidemic	6,731	Swine fever–epidemic	23,919	Swine fever–epidemic	30,621
Swine fever–prevention and control	5,110	Agriculture–Ministry of Rural Affairs	9,896	Agriculture–Ministry of Rural Affairs	14,091
Prevention and control–work	4,305	Occur–Africa	8,013	Swine fever–virus	12,082
Swine fever–virus	4,146	Obtain–effective	4,592	Swine fever–prevention and control	10,805
Agriculture–Ministry of Rural Affairs	2,868	Harmless–dispose	3,807	Prevention and control–work	8,286
Animal–epidemic disease	1785	Animal–epidemic disease	3,766	Occur–Africa	7,334
Detection–Africa	1,394	China–animal	3,334	Animal–epidemic disease	6,423
Occur–Africa	1,314	Effective–control	3,160	Harmless–dispose	5,044
Shuanghui–respond	1,213	Anima--health	2,840	Detection–Africa	4,462
  As can be seen, the concerns of the three user groups were similar, with semantic association co-occurrence phrases such as “Africa--swine fever,” “swine fever--epidemic” and “agriculture--Ministry of Rural Affairs” being common to all users and having a co-occurrence frequency greater than 2,800. This indicated that all users were focused on the ASF epidemic development and had paid attention to the ASF response and treatment measures by the Agricultural Ministry of Rural Affairs. It is worth noting that the viral spread of online information has led to the widespread monitoring of government actions, which puts enormous pressure on the government to respond swiftly. However, there were some focus differences between the three user groups; for example, the co-occurrence phrase “Shuanghui-- respond” was on the list for the verified individual users, which indicated that these users were concerned about the swine fever virus that had been detected in Shuanghui ham, a common food, and were paying close attention to the Shuanghui Group responses. However, institutional users and ordinary users appeared to be more concerned about the treatment of the pigs infected with ASF as evidenced in the co-occurrence phrase “harmless--dispose,” which, respectively, appeared over 3,800 and 5,000 times.
2. Visual analysis of the semantic association
  To illustrate the concern differences in the three user groups more intuitively, semantic association visualizations were drawn, as shown in Figure 6, in which the nodes represent the keywords the users had paid attention to: the larger and darker the node, the higher the concern; with the edges representing the connections between the two words: the thicker and darker the edge, the more frequent the co-occurrence of phrases, and the stronger the connection.
  **Figure 6:**
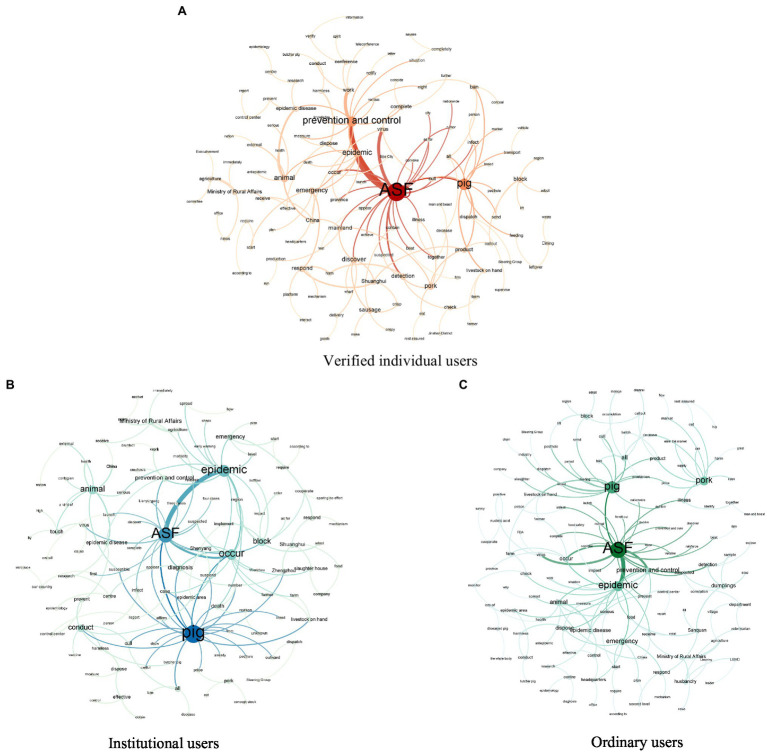
Semantic association visualization for three types of users.
  From the discussions similarity among the three user groups, “ASF” occupied a core position in the semantic association graph, indicating that ASF had received a lot of attention from online users. The risk and uncertainty of a public health emergency may cause changes in public mood and behavior, causing unnecessary fear and anxiety, and thus triggering widespread discussion. The Weibo openness, communication and immediacy extended such events communication efficiency and audience channels (Yu et al., 2017). Another common concern among the three user groups was the “pig.” This is because pork accounts for a large portion of people’s daily diet (about two-thirds of the total meat consumption; Xie et al., 2020), and the pigs health and the pork safety had aroused widespread public concern. Another important topic associated with “ASF” was “epidemic,” which was similar to the research results in Xie et al. (2021). Because the social healthy development and economic life is closely related to every public, the ASF outbreak significant impact cannot be ignored. Controlling epidemic developments is fundamental to reducing the spread of rumors and reassuring the people. Therefore, it is urgent for relevant institutions to actively take effective measures to deal with the epidemic development.
  From the discussion differences among the three user groups, as can be seen from Figure 6A, the “prevention and control” node was the second largest and strongly associated with “ASF,” which indicated that the verified individual users were most concerned about the prevention and control of ASF and hoped that the relevant institutions would take measures to eliminate or reduce the harm and spread of ASF. Xia et al. (2022) also found that the “epidemic prevention” topic received a lot of attention during the COVID-19 pandemic. Different from the verified individual users concern, “pig” was the core section concern of the institutional users, as shown in Figure 6B. The core nodes associated with “pig” were “number,” “death,” “diagnosis” and “morbidity,” which indicated that the institutional users were more concerned with the swine fever virus diagnosis, morbidity and death rate. This may have been because as the ASF resulted in significant losses to the pig industry, the government, the media and businesses were deeply concerned and were closely following the developments. As shown in Figure 6C, the keywords the ordinary user group most paid attention to were scattered. The main phrases associated with “pig” included “pig--breed,” “pig--product,” “pig--price,” and “cull—pig,” and the keywords associated with “pork” were mainly “price,” “product,” and “eat,” which indicated that the ordinary users were concerned about livelihood issues such as the sharp rise in pork prices caused by ASF and the safety of pork products. The small community composed of “Sanquan--dumplings,” “Sanquan--respond,” “dumplings--suspected,” and “Sanquan--food” was a new focus in the visualization network diagram for the ordinary users. This was because the detection of ASF in Sanquan dumplings resulted in a heated discussion by Weibo users. As Sanquan dumplings are widely sold across China and are eaten by ordinary users, it is not surprising that they paid significant attention to the event.

#### Evolution of User Emotions: Sentiment Analysis

**Figure 7:**
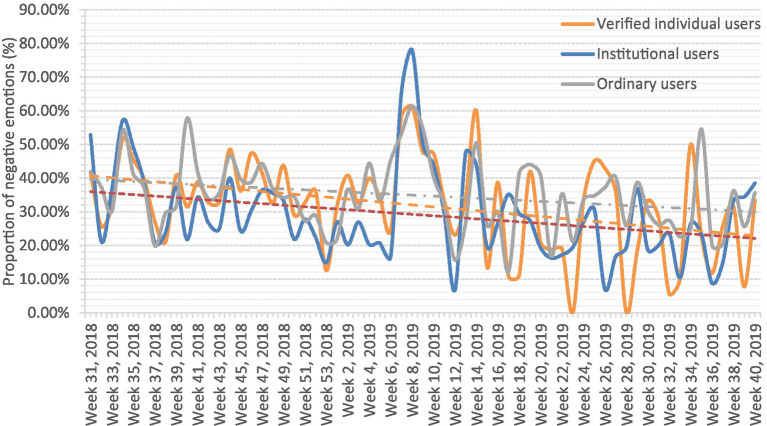
Negative emotion trends.

Although subject analysis can identify the different user groups’ focus, it cannot reveal the different user groups emotional evolution trend, leading to the failure to effectively guide the online public opinion development. Therefore, after counting the sentiment analysis results over time, the ratio of negative emotion posts to all emotional posts from the different user groups were calculated and are shown in Figure 7, in which the horizontal axis represents time, the vertical axis represents the proportion of negative emotions, and the dotted line represents the negative emotion trends for the different user groups.

Over time, the negative emotions in all three user groups had a downward trend, which was similar to the research results in previous studies (Liu et al., 2022; Yang et al., 2022). With the implementation of prevention and control measures and the popularization of knowledge related to the epidemic, people’s fear was also being alleviated. The institutional users tended to have a lower proportion of negative emotions, possibly because they had access to the government agencies that were releasing the often positive and neutral messages. As the verified individual users tended to have fan bases and were opinion leaders in various fields of interest, although the proportion of negative emotions was high at the beginning of the outbreak, it decreased to a level similar to the institutional users as greater attention was being paid to response measures. However, due to the ordinary users’ varying knowledge and education, they tended to express their views only in relation to their own feelings, which meant there was a higher proportion of negative emotions (Vijaykumar et al., 2018).

### Network Structure of the Users Participating in the Discussion: Social Network Analysis

The network analysis was focused on the following three research questions: (1) What was the structure of the ASF information dissemination network? (2) Which users were the main network opinion leaders? and (3) Which users were the network communication bridges?

#### Characteristics of the ASF Information Dissemination Network

To grasp the transmission characteristics of ASF online public opinion network as a whole, the overall network structure index was adopted. The original Weibo posters and their re-posters were imported into the social network analysis tool Gephi, and a network that had 33,666 nodes and 33,114 edges obtained, in which the nodes represented the users who sent out or received a reply, and the edges represented the relationships between those users. On this basis, the statistical function was used to calculate the average path length and network diameter. The average path length is used to measure the network connectivity. If it is less than 6, the network is considered to be closely connected. The results showed that the average path length was 1.47, which is significantly smaller than the boundary value 6, which indicated that the participants were about 1.5 nodes away from each other, which suggested that the participants discussing these themes were very close knit and had close professional proximity. The network diameter, which is the longest distance between any two nodes in the network, was used to measure the network public opinion influence. The network diameter was less than 11, indicating that the public opinion spread was wider and the impact was larger. The community detection method embedded in Gephi was used (Blondel et al., 2008) to reveal that there were more than 1,000 communities in the network, most of which were small. There were three large communities identified: the largest was the “headlines” community, with 39.23% of total users; the second largest was the “People’s Daily,” with 14.93% of total users; and the third largest was the “Xinhua viewpoint,” with 4.22% of users. To further analyze each user role in the public opinion communication network, each user was classified as either “News,” “Government official,” “Organization,” “Leader,” “Interest,” “Public” and “Unknown,” which were defined as in the following.

- News (N): news and media
- Government official (G): official news release platform
- Organization (O): official microblog of organization/institution
- Leader (L): elites of various communities
- Interest (I): Users focused on specific areas of interest
- Public (P): Individual Weibo users
- Unknown (UNK): Account closed or not accessible to retrieve the information

#### Opinion Leaders

In public opinion dissemination networks, the more reposted a user’s views, the more the user is recognized, and the more the user is being seen as an opinion leader in the public opinion network. Identifying opinion leaders in the public opinion communication network is conducive to guiding the public opinion development. Degree has often been used to measure the importance of a node in social network analysis (Hansen et al., 2011). The higher the degree, the more relationships a node has, the more it is in the network core position. Therefore, it is reasonable that degree was used to identify opinion leaders in public opinion communication network. As the large number of nodes and the out-degree of most nodes was 0, for a better visual representation, only the nodes with greater than 10 out-degrees are shown, with those having a greater than 100 out-degrees being marked (Table 5). The two most conspicuous nodes were “N 1” and “N 2,” which were media that had a huge fan base. Media were the most popular sources of information, followed by government and health agencies (Chew and Eysenbach, 2010; Mcneill et al., 2016; Guidry et al., 2017; Vijaykumar et al., 2018; Hellsten et al., 2019). Zhang et al. (2021) reached a similar conclusion, and Zhao et al. (2022b) also proved the opinion leader role in public opinion communication. In addition to the institutional users, the elites from various communities, such as the hosts, lawyers and food safety personnel, also act as opinion leaders in public opinion dissemination, most of which have certain fan bases and social reputations, which meant that their views are more likely to be recognized by ordinary users.

**Table 5:** Users with out-degrees greater than 100.

	Users	Out-degree
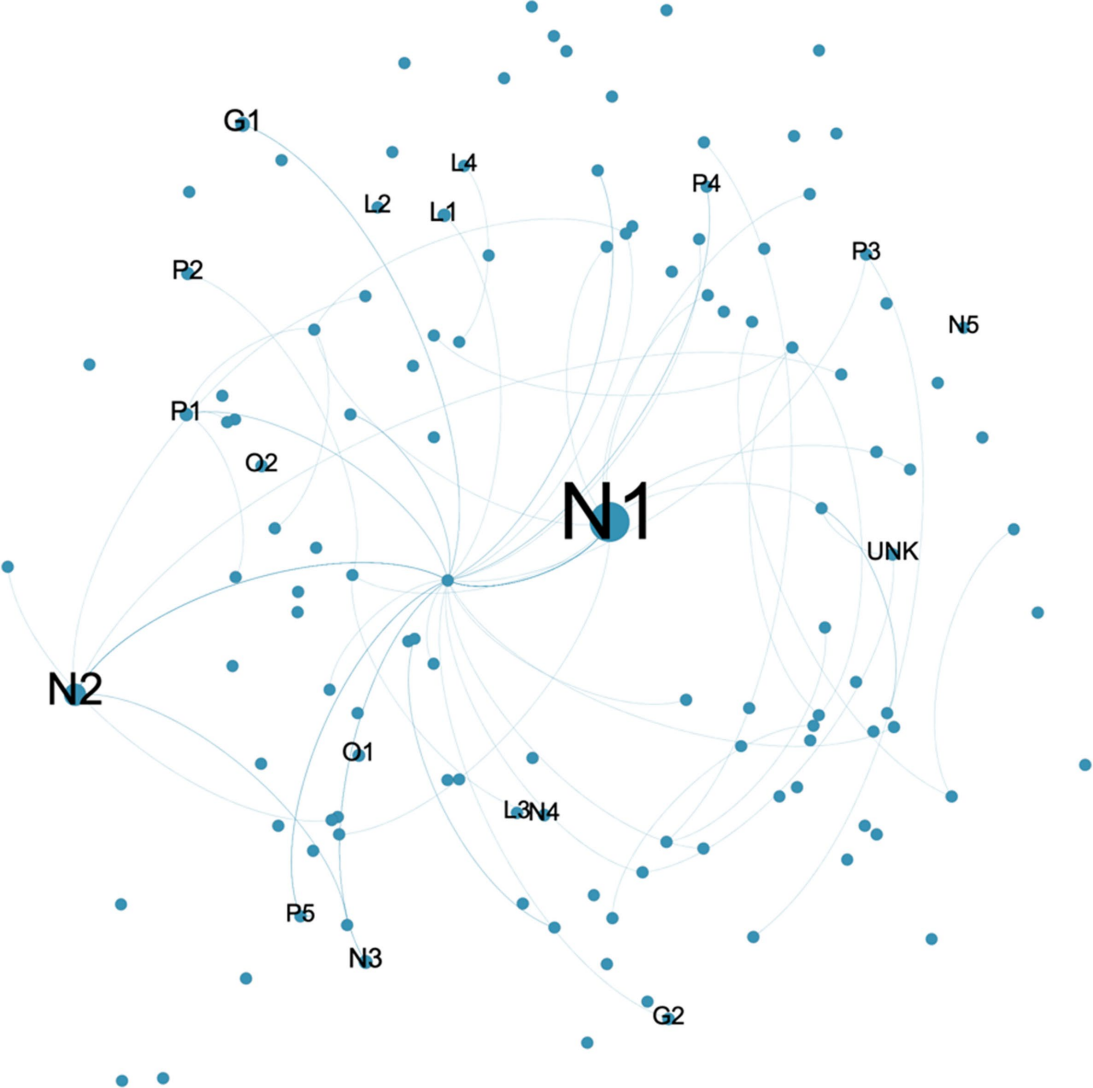	N1	13,468
	N2	5,000
	G1	1,397
	N3	794
	L1	651
	P1	641
	P2	552
	O1	388
	N4	270
	O2	250
	UNK	228
	L2	222
	G2	206
	P3	158
	P4	144
	P5	133
	L3	124
	L4	120
	N5	113

#### Communication Bridges

In public opinion dissemination networks, if the information needs to be transmitted to other users through some users, then these users act as network bridges or gatekeepers. To effectively identify the communication bridge in the network is conducive to controlling the public opinion trend. Betweenness centrality is often used to measure the ability of a node to control the communication between other nodes (Freeman, 1977; Oliveira and Gama, 2012). The higher the value is, the more obvious the bridge role is, so it was used to measure the bridge role in public opinion network. Similar to the opinion leader identification, nodes with a betweenness centrality greater than 10 are shown, and those greater than 100 marked in Table 6. The study of these nodes showed that star fans played very important roles in the dissemination of public opinion as they often paid attention to the Weibo messages and received the news very quickly. Besides, these star fans usually have a certain fan base and are the leaders in their fan group, other fans obtain relevant information through these leaders and then express their own opinions. Bloggers in different fields with a certain fan base and influential celebrities were also found to be the main information disseminators. People come together because of the same interests in such areas as movies, cartoons, and animals. When elites from different fields paid close attention to ASF events, they would publicly express their opinions, and fans would often agree with others’ views by reposting their posts, which further elevates their status as significant information diffusers.

**Table 6:** Users with betweenness centralities greater than 100.

	Users	Value	Users	Value	Users	Value
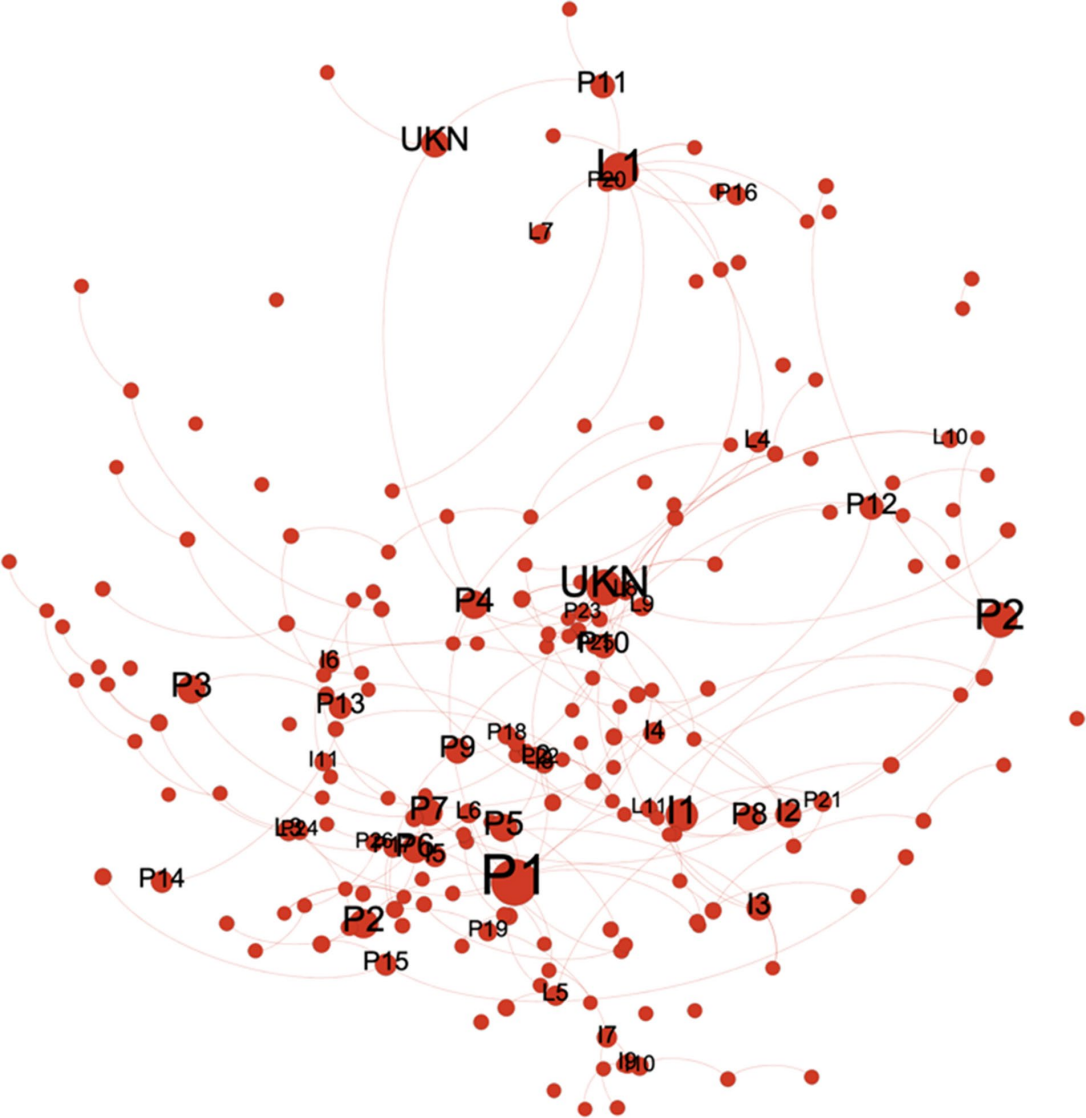	P1	900	P11	285	I9	150
	L1	643	P12	279	L8	140
	UKN	607	P13	259	P17	139
	P2	579	L2	231	P18	138
	I1	509	P14	224	P19	135
	P2	420	I4	219	L9	132
	P3	396	P15	215	I10	128
	P4	395	I5	206	I11	121
	P5	380	L3	198	P20	120
	UKN	378	I6	184	P21	118
	P6	364	L4	180	L10	107
	P7	360	L5	170	P22	106
	I2	330	I7	168	P23	105
	P8	308	P16	161	L11	104
	P9	308	L6	160	P24	104
	I3	297	I8	158	P25	102
	P10	288	L7	156	P26	101

## Conclusions and Recommendations

In this study, content analysis was employed to analyze the online public opinions of different Weibo user groups during the “African swine fever” incident, and network analysis was used to analyze the online public opinion network dissemination characteristics, which contributed to the research field in three ways. First, automatic coding was applied using information mining on the Weibo ASF related users. Second, automatic content analysis methods: topic analysis, semantic network analysis, and sentiment analysis: were applied to study the ASF discussion developments in the three user groups and comparative analysis (three-user groups) of ASF with avian influenza in 2017(content analysis). Third, a network analysis method was applied to study the ASF public opinion network dissemination characteristics and identify the opinion leaders and dissemination bridges.

The following conclusions about the social media discussion on agricultural product safety incidents were made.

Generally speaking, the first occurrence place of agricultural product safety incident and the origin of related agricultural products generated the most intense discussions on the event, and there were more male commenters than female.

The subject word analysis found that the epidemic itself received the highest attention among the four topics, and the prevention topic gained less attention than the disposal and effect topic, which was different from other agricultural product safety incidents (such as avian influenza) that may infect people, which also showed that the dissemination of correct information was fast and effective. The semantic association analysis showed that all three user groups were very concerned about the epidemic development and the response and treatment measures being instigated by the Ministry of Agriculture and Rural Affairs. The verified individual users were most concerned about the prevention and control of the epidemic and play a supervisory role, the content discussed by the institutional users had a high degree of similarity, with the greatest concern being issues related to agricultural production such as morbidity and mortality. In addition to the prevention and control of the epidemic, the ordinary users were also very concerned about the price fluctuations of related agricultural products. What is more, both verified individual users and ordinary users were very concerned about food safety issues, the detection of a virus in the food would deal a serious blow to the brand, leading to the decline of consumer confidence. The emotional polarity found that the proportion of negative emotions was highest in the ordinary user group, decreased over time, and did not exceed 50%.

The network analysis found that media was the main sources of information about agricultural product safety incidents, and only a small amount of the information came from the government and public health institution. Presenters, lawyers and other elites from all walks of life, especially the food safety personnel acted as opinion leaders in the public opinion dissemination. Celebrity fans and bloggers with a certain fan base in different fields of interest such as movies and pets were also found to play a very important role in the dissemination of public opinion.

Therefore, from these results, the following recommendations are given.

First, it is suggested to determine the nature of the agricultural product safety incidents. When an agricultural product safety incident occurs, it is necessary to first make clear whether the consumption of agricultural products with virus will lead to human infection, and timely transmit the results to the public so that they can find a balance between a certain sense of tension and excessive worry. At the same time, information about the measures being taken by the relevant institutions (such as Ministry of Agriculture and Rural Affairs) should be released quickly to gain public trust.

Second, it is advised to take measures to stabilize prices of related agricultural products. Specifically, subsidies and compensations need to be provided to farmers/farms hit by the epidemic, so that they can survive the crisis; agricultural product reserve should be released to avoid a sustained rise in prices caused by demand exceeding supply. In addition, enterprises that use related agricultural products as raw materials should conduct strict quality checks to avoid losing public trust as results of agricultural product safety incidents.

Third, it is suggested to pay attention to the information dissemination role of institutions related to agricultural product safety incidents. When agricultural product safety incidents occur, the public gets most of the latest news from the media since institutions related to agricultural product safety incidents (such as the Ministry of Agriculture and Rural Affairs, public health organizations) own only several social media accounts and much fewer followers than major online media accounts. Therefore, it is necessary to spend effort on the daily operation of these accounts, so that they can become a two-way communication place for obtaining official news and receiving public opinions when agricultural product safety incidents occur. What is more, because the public tends to believe and agree with opinion leaders related to agricultural product safety incidents (such as food safety personnel), they can be used to correctly guide the discussion atmosphere.

The study has the following limitations. First, the analysis of social media discussions of different types of users was built on the basis of information collected about the senders of Weibo. Although users were classified as institutional users, certified individual users, and ordinary users, the definition of their identities was not very accurate because this certification was submitted for modification by users themselves on the basis of certain thresholds, e.g., certified individual users do not necessarily have more influence than ordinary users. Second, in the collection of repost relationships of Weibo users, due to the large span of the study period and the huge number of Weibo posts, they were divided into high, medium, and low heat periods, from which a social network based on repost relationships was established by random sampling, respectively. Such an approach only included a small portion of Weibo reposts, and thus may produce omissions in the subsequent analysis of key users of the spread of agricultural safety events through the network. Future research can address the above issues and improve the accuracy of data collection to obtain social media discussion results that are closer to the reality.

## Data Availability Statement

The original contributions presented in the study are included in the article/supplementary material further inquiries can be directed to the corresponding author.

## Author Contributions

YL: conceptualization, funding acquisition, and methodology QJ: data curation and software. QJ and YX: formal analysis, and writing—original draft. YH and YL: supervision and writing—review and editing. All authors contributed to the article and approved the submitted version.

## Funding

This work was funded by: National Social Science Fund Project of China, grant number 20XZS020; Scientific Research Innovation Team Project of Neijiang Normal University, grant number 2021TD01; and Scientific Research Project of Tuojiang River Basin High-quality Development Research Center, grant number TJGZL2021-06.

## Conflict of Interest

The authors declare that the research was conducted in the absence of any commercial or financial relationships that could be construed as a potential conflict of interest.

## Publisher’s Note

All claims expressed in this article are solely those of the authors and do not necessarily represent those of their affiliated organizations, or those of the publisher, the editors and the reviewers. Any product that may be evaluated in this article, or claim that may be made by its manufacturer, is not guaranteed or endorsed by the publisher.
